# Rumen Metagenomic and Muscle Metabolomic Characterization of Meat Quality in Duolang Sheep at Different Ages

**DOI:** 10.3390/foods15071158

**Published:** 2026-03-30

**Authors:** Xuanyue Li, Yan Li, Qianyi Li, Yaxing Jin, Yong Chen

**Affiliations:** 1 Research Center for Biofeed and Animal Gut Health, College of Animal Sciences, Xinjiang Agricultural University, Urumqi 830052, China; xjaulxy@126.com (X.L.); yanlee@xjau.edu.cn (Y.L.); xjaulqy@163.com (Q.L.); xjaujyx2026@163.com (Y.J.); 2 Xinjiang Herbivore Nutrition Laboratory for Meat & Milk, Xinjiang Agricultural University, Urmuqi 830052, China

**Keywords:** Duolang sheep, meat quality, rumen microbiota, metagenome, metabolome

## Abstract

This study aimed to investigate the changes in the meat quality characteristics of Duolang sheep using rumen metagenomic and muscle metabolomic analyses across different age groups. A total of 24 three-month-old male Duolang sheep were selected and reared, and samples of *longissimus thoracis* muscle and rumen contents were collected at 4, 6, and 8 months of age to evaluate meat quality, metabolites, rumen metagenome, and volatile fatty acids (VFAs). The results indicated that the lightness (L*_45min_) and yellowness (b*_45min_) of the *longissimus thoracis* muscle at 45 min post-slaughter were significantly higher at 4 and 6 months than at 8 months of age (*p* < 0.05). In terms of ruminal VFAs, butyrate concentration was significantly higher at 6 months than at 4 months (*p* < 0.05), and valerate concentration exhibited a quadratic relationship with age (*p* = 0.02). With increasing age, the relative abundances of *Prevotella* and *Fibrobacter* increased, whereas those of *Methanobrevibacter* and Bacteroides decreased (*p* < 0.05), leading to shifts in functional pathways related to amino acid, lipid, and carbohydrate and energy metabolism. Untargeted metabolomics revealed that muscle betaine and inosine peaked at 4 months of age, whereas L-arginine, L-proline, and inosinic acid were most abundant at 6 months of age (*p* < 0.05). Correlation analysis revealed that the b*_45min_ was positively associated with ruminal concentrations of propionate, butyrate, and valerate, as well as with the relative abundances of key Selenomonadales taxa (*p* < 0.05). Inosinic acid exhibited a positive correlation with the abundance of the genus *Sodaliphilus* and ruminal butyrate concentration (*p* < 0.05), while *Sodaliphilus* abundance was negatively correlated with inosine (*p* < 0.05). In summary, this study demonstrates that age-related variations in the meat quality of Duolang sheep are closely associated with rumen microbial ecology and muscle metabolites, offering novel insights into the molecular mechanisms underlying meat quality formation and identifying potential biomarkers.

## 1. Introduction

Meat is an essential component of the human diet and a critical source of high-quality dietary protein. Lamb is valued for its high nutritional content, being abundant in essential amino acids, fatty acids, and other bioactive compounds [1]. Unlike other animal products, lamb is characterized by its distinct sensory properties, particularly its unique volatile compounds and flavor profile, which have garnered significant consumer preference. Meat quality is a complex phenotypic trait influenced by multiple factors such as diet [2], breed [3], age [4], and sex [5]. Studies have shown that 12-month-old Hetian white lambs exhibit higher contents of amino acids, proteins, and free fatty acids compared to 6-month-old lambs [4]. Additionally, male lambs have demonstrated greater fat deposition capacity and antioxidant ability than females [4]. Han et al. observed dynamic changes in lipid deposition across different growth stages in Sunit sheep, with notable differences in subcutaneous fat between early and late developmental phases [6].

Ruminants are capable of converting indigestible macromolecules into livestock products (meat and milk) for human consumption through the rumen, where the composition, structure, and function of the rumen microbiota play a crucial role in nutrient digestion, absorption, and product conversion [7]. The gut–muscle axis has been proposed to describe the interactions between gut microorganisms and host muscle tissues. While the gut–muscle axis [8] and brain–muscle axis [9] have been well documented in human studies, they remain less explored in ruminants. The gastrointestinal tract produces a range of bioactive compounds that can directly influence the brain [10] and muscles [11]. Previous research has shown that the composition and metabolic profile of sheep muscle vary across developmental stages [12]. Specifically, fatty acids derived from microbial metabolism promote fat deposition, while volatile fatty acids (VFAs) serve as key mediators of muscle metabolism, regulating adipocyte differentiation and triglyceride synthesis [13]. Given the essential role of rumen microbiota and their metabolites in ruminant digestive physiology, this study hypothesizes that the diversity, structure, and function of rumen microbiota in ruminants change with age, coupled with shifts in their metabolic outputs, modulate muscle quality and metabolite profiles through the gut–muscle axis.

Duolang sheep is a local breed from southern Xinjiang, China, renowned for its superior meat quality. However, systematic studies on meat quality differences across ages or growth stages in this breed remain limited. In this study, Duolang rams of different ages were selected as a research model to evaluate meat quality parameters of the *longissimus thoracis* muscle and VFA contents in the rumen. Metagenomic technology was employed to investigate differences in rumen microbial composition and function among Duolang sheep of different ages, while liquid chromatography–mass spectrometry (LC–MS)-based untargeted metabolomics was used to analyze variations in muscle metabolites. This work comprehensively explores the rumen metagenomic and muscle metabolomic characteristics associated with meat quality, providing theoretical insights for axial theory applications in livestock production and meat quality regulation, as well as novel perspectives for enhancing the meat quality of Duolang sheep.

## 2. Materials and Methods

### 2.1. Animals, Experiment Design, and Feeding Management

The animal feeding trial was conducted at the breeding base in Bashi Airike Village, Awat County (Aksu, Xinjiang, China), from May to September 2023. Prior to the trial, sheep pens and feeding troughs were thoroughly cleaned and disinfected. The number of animals was determined based on the report by Zhang et al. and the principle of minimizing animal use [14]. A total of 24 healthy, three-month-old, unrelated male Duolang lambs with similar body weights and birth dates were purchased from Aksu Fumin Livestock Breeding Co., Ltd. (Aksu, Xinjiang, China). Lambs were randomly divided into three groups (*n* = 8 per group), with each lamb serving as an independent experimental unit. All animals were housed in individual pens under uniform environmental conditions. The three groups were raised until 4, 6, and 8 months of age, respectively, at which point the trial concluded and the animals were slaughtered. The basal diet was formulated according to the Nutrient Requirements of Meat-type Sheep and Goat (NY/T 816-2021) [15]. The composition and nutrient levels of the basal diet are shown in Table 1. During the trial, the sheep were fed twice daily at 10:00 and 19:00 with *ad libitum* access to feed and water. Deworming, vaccination, and general management followed the Technical Specification for Feeding and Management of Stall Feeding Meat-producing Sheep and Goats (NY/T 3052-2016) to ensure animal welfare [16].

### 2.2. Sampling

Sampling was conducted in a random order to avoid time effects. On the first day after the feeding trial concluded, sheep were fasted for 24 h with water withheld for the final 8 h, and then humanely slaughtered at a commercial abattoir (Xiyushayuan Co., Ltd., Awati, Xinjiang, China). The *longissimus thoracis muscles* between the 12th and 13th ribs from the left side of each carcass were excised. Each muscle sample was divided into two portions: one for measuring pH, meat color, shear force, and drip loss, and the other immediately flash-frozen in liquid nitrogen for subsequent metabolomic analysis. Concurrently, rumen contents were collected and immediately stored at −20 °C for subsequent metagenomic sequencing and VFAs analysis.

### 2.3. Meat Quality

Shear force was determined 45 min after slaughter: fresh muscle samples were heated in an 80 °C water bath until the internal temperature reached 70 °C, then removed and cooled to room temperature (20 °C). Subsequently, cylindrical core samples were taken parallel to the muscle fiber orientation to obtain standardized specimens (2 cm × 3 cm × 5 cm). Measurements were performed using a C-LM3B digital tenderness meter (Tenovo, Beijing, China) with a 25 kg load cell and a crosshead speed of 5 mm/s. Each sample was measured in triplicate, and the average value was calculated. Shear force was reported in kilograms of force (kgf). pH measurements were taken at 45 min and 24 h post-slaughter using a pH-Star meter (Matthäus, Beilin, Germany) with built-in temperature compensation. Meat color was assessed using a CR-10 chroma meter (Konica Minolta, Osaka, Japan) at three randomly selected locations on the muscle surface, and the colorimetric parameters *L*, a*, and b* were recorded. The aperture, light source, and observation angle were set as illuminant D65, 8 mm, and CIE10°, respectively. For drip loss, trimmed meat samples (50 mm × 30 mm × 20 mm) were individually sealed in custom air-filled bags and refrigerated at 4 °C for 24 h [17].

Drip loss (%) = [(initial weight (g) − final weight (g))/initial weight (g)] × 100

### 2.4. Ruminal Volatile Fatty Acids

VFAs in rumen contents were determined using a gas chromatograph (GC-2010; Shimadzu, Kyoto, Japan) equipped with a Stabilwax DA column (Restek, Bellefonte, PA, USA). Nitrogen was used as the carrier gas, and 4-methylvaleric acid served as the internal standard. The chromatographic conditions were as follows: injector temperature, 230 °C; column oven temperature programmed from an initial 55 °C to 200 °C at a rate of 13 °C/min and held for 0.5 min; flame ionization detector (FID) temperature, 240 °C [18].

### 2.5. Metagenomic Sequencing of Rumen Microorganisms

#### 2.5.1. DNA Extraction and Sequencing

Genomic DNA from rumen microorganisms was extracted using the QIAamp DNA Mini Kit (Qiagen, Hilden, Germany). DNA concentration, integrity, and purity were assessed with an Agilent 5400 system (Agilent, Santa Clara, CA, USA). Libraries were constructed using the NEB Next^®^ Ultra™ DNA Library Prep Kit for Illumina^®^ (New England Biolabs, Ipswich, MA, USA). Qualified DNA samples were randomly fragmented to approximately 350 bp using an ultrasonicator (Covaris, Woburn, MA, USA). The DNA fragments were then subjected to end repair, polyA tailing, adapter ligation, purification, and PCR amplification to complete library preparation. Finally, the PCR products were purified with the AMPure XP system (Beckman Coulter, Brea, CA, USA), the library insert size was verified using a Bioanalyzer (Agilent 2100; Agilent Technologies, Santa Clara, CA, USA), and library concentration was quantified by means of real–time PCR. Sequencing was carried out on a high-throughput sequencing platform (PE150; Illumina, San Diego, CA, USA), generating 150 bp paired-end reads.

#### 2.5.2. Metagenomic Data Quality Control, Assembly, and Coding Sequence Gene Prediction

Raw sequencing data were processed using KneadData (v0.7.4) for quality control and Bowtie2 for host DNA removal. FastQC (v0.11.9) was employed to evaluate data quality to ensure the validity and effectiveness of the procedures [19,20]. Host-depleted clean reads from all samples were assembled into contigs using MEGAHIT (v1.2.9) [21]. Open reading frames (ORFs) were predicted from these contigs using Prodigal (v2.6.3). Non-redundant gene sets were generated by clustering and removing redundant sequences with CD-HIT (v4.8.1). Gene abundance was quantified using Salmon (v1.10.2). For functional annotation, the non-redundant genes were aligned against multiple databases using eggNOG-mapper (v2.0.1) and DIAMOND (v0.8.22), and corresponding relative abundance profiles were generated for each database [22,23,24,25]. Taxonomic annotation was performed by aligning non-redundant protein sequences to the NCBI NR protein database using DIAMOND (v0.8.22), with taxonomic assignments carried out using BASTA (v1.4.1). Meanwhile, species-level sequence counts were estimated using Kraken2 in conjunction with a custom microbial nucleotide database (comprising bacterial, fungal, archaeal, and viral sequences filtered from the NCBI NT and RefSeq whole–genome databases). These estimates were subsequently refined using Bracken (v2.0) to obtain accurate species abundance profiles [26,27,28,29].

#### 2.5.3. Gene and Pathway Abundance Analysis

High-quality reads were aligned to the non-redundant gene catalog with a 95% identity threshold using SOAPaligner (v2.22), and gene counts per sample were quantified. Functional annotation of the genes was performed by comparing the non-redundant protein sequences against the following protein databases: the Kyoto Encyclopedia of Genes and Genomes (KEGG; http://www.genome.jp/kegg/ (accessed on 20 December 2024)) and the Carbohydrate–Active enZYmes Database (CAZy; https://www.cazy.org/ (accessed on 20 December 2024)).

### 2.6. Untargeted Metabolomics of Longissimus Thoracis Muscle

#### 2.6.1. Metabolites Extraction

A 50-mg portion of the meat sample was weighed and mixed with 1000 µL of extraction solvent (methanol: acetonitrile: water = 2:2:1, *v*/*v*/*v*) containing an internal standard (final concentration: 20 mg/L) by vortexing for 30 s. Steel beads were added, and the mixture was processed in a grinder at 45 Hz for 10 min, followed by ultrasonication (40 kHz) for 10 min in an ice-water bath and subsequent incubation at −20 °C for 1 h. The sample was then centrifuged at 4 °C and 12,000× *g* for 15 min. Subsequently, 500 µL of the supernatant was transferred into a new microcentrifuge tube. The extract was dried in a vacuum concentrator. The dried metabolites were reconstituted with 160 µL of reconstitution solvent (acetonitrile:water = 1:1, *v*/*v*), vortexed for 30 s, and ultrasonicated in an ice-water bath for 10 min. The sample was then centrifuged again at 4 °C and 12,000× *g* for 15 min. Finally, 120 µL of the supernatant was transferred into a 2-mL injection vial. For quality control, 10 µL from each sample was pooled to create a QC sample for instrumental analysis.

#### 2.6.2. Liquid Chromatography-Tandem Mass Spectrometry (LC-MS/MS)

Metabolomics analysis was performed using an ultra-high-performance liquid chromatography (UPLC) system (ACQUITY UPLC I-Class PLUS; Waters Corp., Milford, MA, USA) coupled with a high-resolution mass spectrometer (MS) (Xevo G2-XS QTOF; Waters Corp., Milford, MA, USA). Chromatographic separation was achieved on an ACQUITY UPLC HSS T3 column (2.1 mm × 100 mm, 1.8 μm; Waters Corp., Milford, MA, USA). Chromatographic separation was performed using a mobile phase system consisting of 0.1% formic acid in water (A) and 0.1% formic acid in acetonitrile (B) for both positive and negative ion modes.

MS data were acquired in MSE mode using the Xevo G2-XS QTOF MS collected by MassLynx software (V4.2; Waters Crop., Milford, MA, USA). In each data acquisition cycle, dual-channel data acquisition was performed on both low collision and high collision energies. The low collision energy was set at 2 V, while the high collision energy was ramped from 10 to 40 V. The scan time was 0.2 s per spectrum. The electrospray ionization (ESI) parameters were optimized as follows: capillary voltage, 2.0 kV (positive mode) or −1.5 kV (negative mode); cone voltage, 30 V; source temperature, 150 °C; desolvation temperature, 500 °C; cone gas flow, 50 L/h; and desolventation gas flow, 800 L/h.

#### 2.6.3. Data Preprocessing, Annotation, and Analysis

Raw data were processed using Progenesis QI software (v2.0; Waters Corp., Milford, MA, USA) for peak detection, peak alignment and integration. Metabolite identification was performed using the online METLIN database (https://metlin.scripps.edu, accessed on 3 January 2024) and Biomark’s proprietary library, with a mass tolerance for both precursors and fragments set at 50 ppm. Peak areas were normalized to the total peak area for downstream analysis. Principal component analysis (PCA) and Spearman correlation analysis were used to assess the repeatability of intra-group and quality control samples. Identified compounds were annotated with classification and pathway information from the KEGG, HMDB and LIPID MAPS databases. Fold change (FC) values were calculated, and Student’s *t*-test was used to determine the statistical significance (*p*-value) of each metabolite. Orthogonal projections to latent structures-discriminant analysis (OPLS-DA) modeling was performed using the ropls package in R (v4.5.0), with model validity confirmed by 200 permutations.

### 2.7. Statistical Analysis

Meat quality, ruminal VFA concentrations, and α-diversity indices of the rumen metagenome were analyzed using one-way analysis of variance (ANOVA) with SPSS Statistics (v26.0; IBM, Armonk, NY, USA). Orthogonal polynomial contrasts were applied to examine linear and quadratic effects of lamb age. Prior to analysis, data normality and homogeneity of variances were verified using the Shapiro-Wilk and Levene’s tests, respectively. Data are presented as mean and standard error of the mean (SEM). Differences in the relative abundance of ruminal microbial species were assessed using the Kruskal–Wallis test. Statistical significance was defined as *p* < 0.05.

For differential metabolite analysis, variable importance in projection (VIP) values were calculated via multiple cross-validation. Differential metabolites were identified based on the criteria of fold change (FC) > 1.0, *p* < 0.05 and VIP > 1.0. KEGG pathway enrichment analysis was conducted using the hypergeometric distribution test. Differences in the relative abundances of microbial carbohydrate-active enzyme genes were assessed using the Kruskal–Wallis test.

The heatmap was analyzed using R (v3.5.1) with the stats package. Correlation analysis was performed using the mantel_test function in the ggcor package (v0.9.8.1) under R (v4.2.0).

## 3. Results

### 3.1. Meat Quality of Longissimus Thoracis Muscle in Duolang Sheep at Different Ages

The meat quality parameters of Duolang sheep at different ages are given in Table 2. In this study, as the age increased, the post-slaughter pH_45min_ showed a significant linear increase (*p* = 0.049), while L*_45min_ and b*_45min_ exhibited significant linear decreases (*p* = 0.02 for both). The L*_45min_ value in the *longissimus thoracis* muscle of 4- and 6-month-old sheep was significantly higher than that of the 8-month-old group (*p* < 0.05). At 24 h post-slaughter, no significant differences were observed in pH, meat color, shear force (tenderness), or drip loss among the groups (*p* > 0.05).

### 3.2. Ruminal VFAs in Duolang Sheep at Different Ages

The VFA profiles in the rumen contents of Duolang sheep at different ages are presented in Table 3. Propionate concentration in the 6-month-old group was higher than that in the 4-month-old group (*p* > 0.05). The butyrate concentration demonstrated a significant linear increase with advancing age (*p* = 0.03). Specifically, the 8-month-old group had a significantly higher concentration than that of the 4-month-old group (*p* < 0.05). The valerate concentration exhibited a quadratic relationship with age (*p* = 0.02), with the 6-month-old group showing a higher value than both the 4- and 8-month-old groups (*p* < 0.05), while the concentration in the 4-month-old group was significantly lower than that in the 8-month-old group (*p* < 0.05) However, no significant differences were observed in the total volatile fatty acid concentration among the groups (*p* > 0.05).

### 3.3. Rumen Metagenome

#### 3.3.1. Composition and α-Diversity of Rumen Microbiota in Duolang Sheep at Different Ages

**Figure 1:**
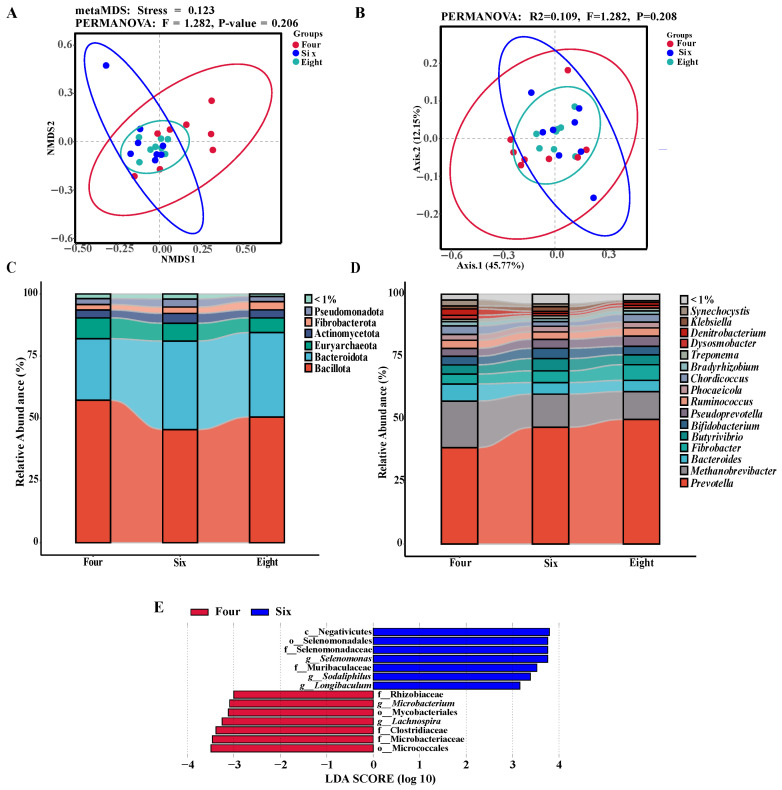
Analysis of rumen metagenomic microbial diversity in Duolang sheep at different ages. (**A**) Non-metric multidimensional scaling (NMDS) based on Bray–Curtis. (**B**) Principal coordinate analysis (PCoA) based on Bray–Curtis. (**C**) Relative abundance of rumen microbiota at the phylum level in Duolang sheep at different ages. (**D**) Relative abundance of rumen microbiota at the genus level in Duolang sheep at different ages. (**E**) Linear discriminant analysis Effect Size (LEfSe) analysis. Four, 4 months of age; Six, 6 months of age; Eight, 8 months of age.

The α-diversity of ruminal microbiota is presented in Table 4. The 6-month-old group exhibited significantly higher ACE and Chao1 indices compared to the 8-month-old group (*p* < 0.05). Non-metric multidimensional scaling (NMDS) and principal coordinate analysis (PCoA) based on Bray–Curtis dissimilarity revealed distinct differences in the overall ruminal microbial composition between the 4-month-old group and the 6- and 8-month-old groups (Figure 1A,B). The composition of the ruminal microbiota at different taxonomic levels was further analyzed. The microbial composition at the phylum level is shown in Figure 1C. Among all groups, Bacillota exhibited the highest relative abundance (50.52%, 57.59%, and 45.62%, respectively), followed by Bacteroidota (33.76%, 24.09%, and 35.28%, respectively). At the genus level (Figure 1D), *Prevotella* showed the highest relative abundance (45.45%, 35.76%, and 42.03%, respectively), followed by *Methanobrevibacter* (10.29%, 17.50%, and 10.72%, respectively). LEfSe analysis revealed that the differentially abundant taxa in the 6-month-old group were the class Negativicutes, order Selenomonadales, families Selenomonadaceae and Muribaculaceae, and genera *Selenomonas*, *Sodaliphilus*, and *Longibaculum* (Figure 1E). The differentially abundant taxa in the 4-month-old group were the class Mycobacteriales, order Micrococcales, families Rhizobiaceae, Clostridiaceae, and Microbacteriaceae, and genera *Microbacterium*, and *Lachnospira*.

#### 3.3.2. Biomarkers of Metabolic Potential in the Rumen Microbiota of Duolang Sheep at Different Ages

**Figure 2:**
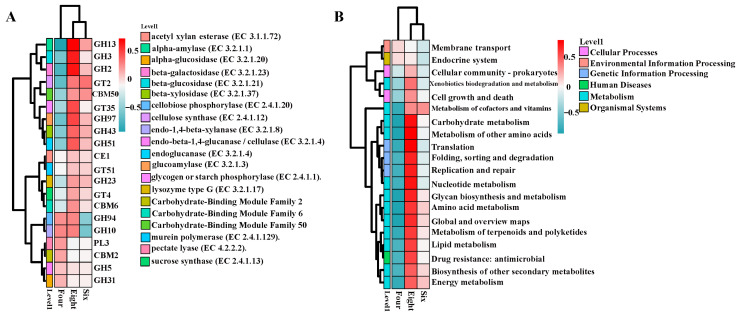
Biomarkers of metabolic potential in the rumen microbiota of Duolang sheep at different ages. (**A**) CAZy carbohydrate metabolic heatmap of rumen microbiota in Duolang sheep across age groups. (**B**) KEGG level 2 functional heatmap of rumen microbiota in Duolang sheep across age groups.

To investigate the functional alterations in the rumen microbiota of Duolang sheep at different ages, the functional composition profiles and differential metabolic pathways were analyzed based on the KEGG database. Clustered heatmap analysis was performed on the functional characteristics of carbohydrate-active enzyme (CAZy) families, including glycoside hydrolases (GHs), glycosyltransferases (GTs), carbohydrate-binding modules (CBMs), and polysaccharide lyases (PLs), which reflect the carbohydrate metabolic potential of the rumen microbial communities across different age groups. As shown in Figure 2A, most GTs and GHs in the 8-month-old group exhibited higher abundance in the 8-month-old group, whereas their abundance was lower in the 4-month-old group. In the functional annotation analysis of the rumen microbiota, the top 20 entries at KEGG Level 2 were summarized, covering six major categories: Cellular Processes, Environmental Information Processing, Genetic Information Processing, Human Diseases, Metabolism, and Organismal Systems. Subsequently, a clustered heatmap analysis of these functions (Figure 2B) revealed that the 8-month-old group showed positive correlations with functions such as amino acid metabolism, carbohydrate metabolism, and lipid metabolism, while the 4-month-old group exhibited negative correlations.

### 3.4. Muscle Untargeted Metabolomics

#### 3.4.1. Metabolic Profile of Longissimus Thoracis Muscle in Duolang Sheep at Different Ages

**Figure 3:**
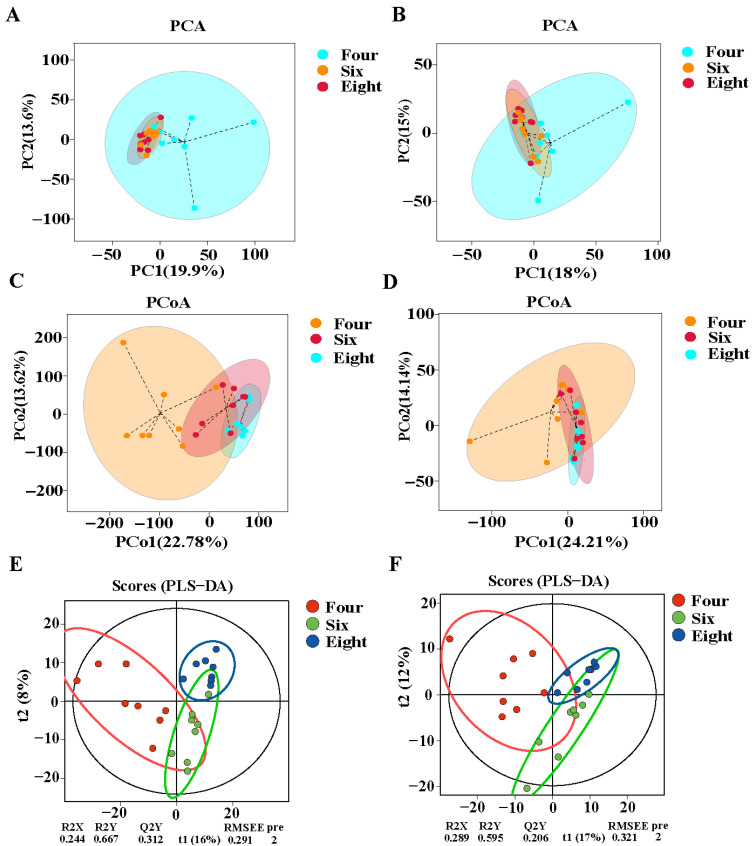
Metabolic profile of *longissimus thoracis* muscle in Duolang sheep at different ages. (**A**) PCA score plot in positive ion mode. (**B**) PCA score plot in negative ion mode. (**C**) PCoA score plot in positive ion mode. (**D**) PCoA score plot in negative ion mode. (**E**) PLS-DA score in positive ion mode. (**F**) PLS-DA score in negative ion mode. Four, 4 months of age; Six, 6 months of age; Eight, 8 months of age.

To investigate changes in the metabolic profiles of the *longissimus thoracis* muscle in Duolang sheep at different ages, this study employed LC-MS-based untargeted metabolomics. Compositional differences among groups were evaluated using principal component analysis (PCA), principal coordinate analysis (PCoA), and partial least squares-discriminant analysis (PLS-DA). As shown in Figure 3, PCA indicated that the metabolites of the three groups did not exhibit clear separation in either positive or negative ion mode (Figure 3A,B). PCoA revealed distinct separation between the 4-month-old group and both the 6- and 8-month-old groups in the positive ion mode, whereas no obvious separation was observed among the three groups in the negative ion mode (Figure 3C,D). PLS-DA demonstrated clear differentiation among the three experimental groups (Figure 3E,F), indicating significant differences in the metabolic profiles of the *longissimus thoracis* muscle of Duolang sheep across different developmental stages.

#### 3.4.2. Differential Metabolites in Longissimus Thoracis Muscle of Duolang Sheep at Different Ages

**Figure 4:**
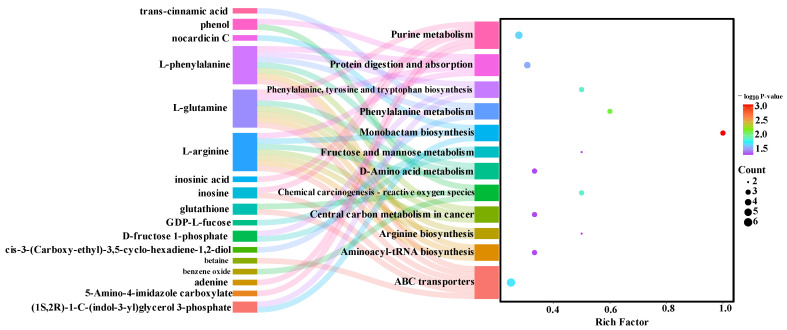
KEGG-based Sankey diagram of differential metabolic pathways: 4 months vs. 6 months of age.

**Figure 5:**
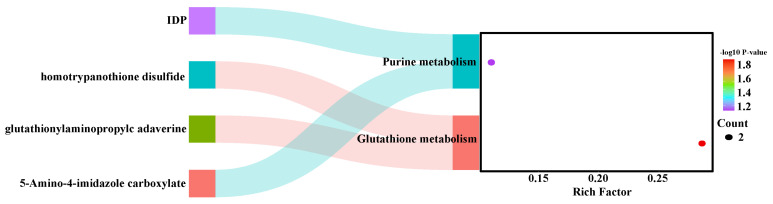
KEGG-based Sankey diagram of differential metabolic pathways: 6 months vs. 8 months of age.

**Figure 6:**
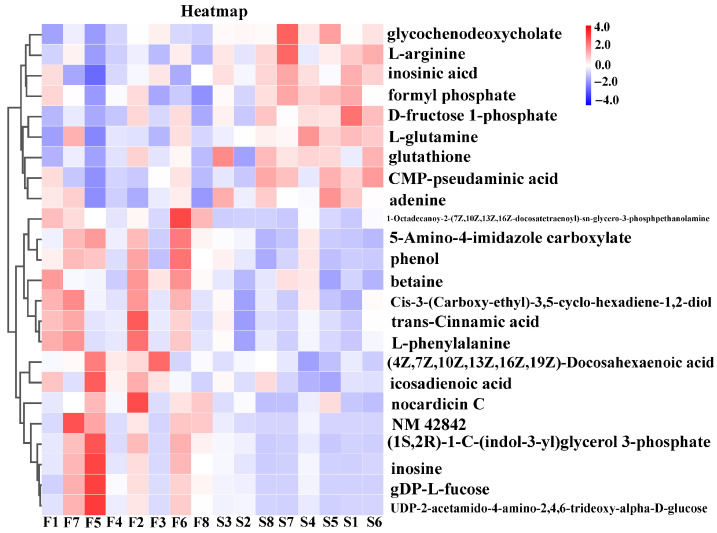
Heatmap of differential metabolites: 4-month-old vs. 6-month-old groups. F, 4-month-old age group; S, 6-month-old age group.

**Figure 7:**
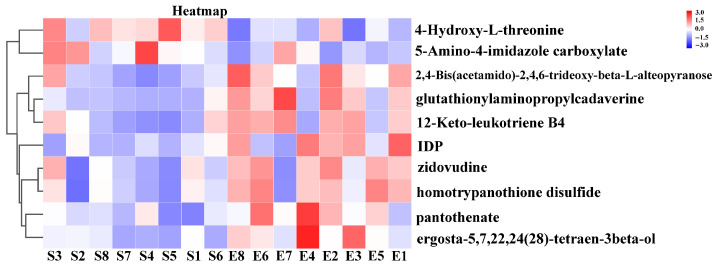
Heatmap of differential metabolites: 6-month-old vs. 8-month-old groups. S, 6-month-old age group, E, 8-month-old age group.

To further investigate the metabolic pathways in the *longissimus thoracis* muscle of Duolang sheep at different ages, we performed KEGG pathway analysis to annotate the involved metabolic routes. The enriched differential pathways between age groups are presented in Figure 4 and Figure 5. The enriched pathways between the 4- and 6-month-old groups included phenylalanine metabolism; phenylalanine, tyrosine, and tryptophan biosynthesis; arginine biosynthesis; alanine, aspartate, and glutamate metabolism; glycan degradation; fructose and mannose metabolism; glyoxylate and dicarboxylate metabolism; protein digestion and absorption; bile secretion; mineral absorption; biosynthesis of unsaturated fatty acids; ABC transporters; purine metabolism; and aminoacyl-tRNA biosynthesis (Figure 4). The enriched pathways between the 6- and 8-month-old groups included purine metabolism and glutathione metabolism (Figure 5). Based on the metabolites identified in these enriched pathways, the heatmap analysis was conducted to visualize the differences between the groups (Figure 6 and Figure 7). It was found that in the comparison between the 4-month-old and the 6-month-old groups, metabolites such as arginine, inosinic acid, and glutamine were upregulated in the 6-month-old group. In the comparison between the 6-month-old and the 8-month-old groups, 4-hydroxy-L-threonine and 5-amino-4-imidazole carboxylate were significantly upregulated in the 6-month-old group.

### 3.5. Correlation Analysis of Differential Microbes, VFAs, Meat Quality, and Differential Metabolites in Longissimus Thoracis Muscle

**Figure 8:**
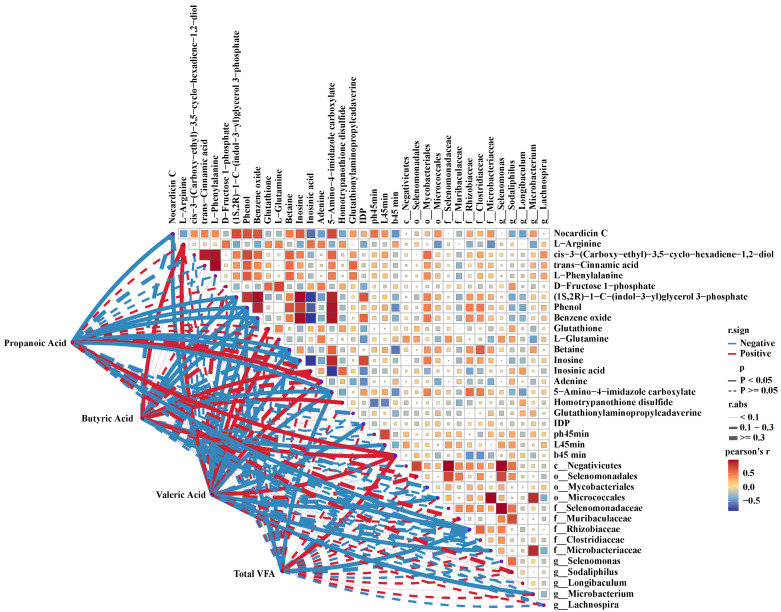
Heatmap of associations among rumen differential microbiota, VFAs, meat quality, and metabolites.

In this study, Pearson correlation analysis was performed to investigate the relationships among differential rumen microbiota, VFAs, meat quality traits, and differential metabolites (Figure 8). The results revealed that the b*_45min_ was significantly positively correlated with ruminal concentrations of propionate, butyrate, and valerate, as well as with the relative abundances of the class Negativicutes, order Selenomonadales, family Selenomonadaceae, and genus *Selenomonas*. Conversely, the L*_45min_ showed a significant negative correlation with ruminal butyrate concentration. Additionally, pH_45min_ was significantly negatively correlated with the relative abundance of the genus *Longibaculum.* Regarding amino acid metabolites, L-arginine exhibited significant positive correlations with the abundances of the order Selenomonadales, family Selenomonadaceae, and genus *Selenomonas*, as well as with the ruminal concentrations of propionate and butyrate. L-phenylalanine was significantly positively correlated with the relative abundance of the genus *Lachnospira*. Similarly, L-glutamine showed significant positive correlations with the abundances of the class Negativicutes, ordor Selenomonadales, family Selenomonadaceae, and genus *Selenomonas*.

## 4. Discussion

Meat color is one of the key factors influencing consumers’ purchasing decisions, as it is primarily determined by myoglobin and its heme content. In this study, the lightness (L*) decreased linearly with increasing age. Additionally, the redness (a*) at 45 min post-slaughter was higher in the 6-month-old group than in the 4-month-old group. These findings are consistent with the results reported by Bakhsh et al. [30]. Previous studies have shown that muscle lightness is related to myoglobin content and pH at 24 h post-slaughter. An increase in myoglobin content reduces the lightness of lamb muscle and darkens the meat, while a low pH weakens the linkage between myoglobin heme and structural proteins, thereby accelerating heme oxidation and leading to color changes [31]. Age significantly affects the proportion of myoglobin in muscle, with the myoglobin content increasing with advancing age. Moreover, the increased heme iron content in muscle tissue with advancing age could contribute to the enhanced redness, which aligns with the outcomes of this study. Therefore, in this study, the increased age of Duolang sheep led to a rise in myoglobin content, resulting in reduced meat lightness.

Rumen bacteria in ruminants are primarily categorized into fiber-, hemicellulose-, lipid-, protein-, and starch-degrading groups, and the composition, structure, and function of the rumen microbiota change with host age [32]. In this study, the dominant phyla were Bacillota (formerly Firmicutes) and Bacteroidota (formerly Bacteroidetes), with *Prevotella* and *Methanobrevibacter* being the predominant genera in the rumen contents of Duolang sheep. Moreover, the diversity and structure of the rumen microbiota changed with increasing age, which is consistent with previous findings [33]. To further investigate the impact of rumen microbiota shifts on meat quality, KEGG-based functional annotation analysis revealed that the 8-month-old group exhibited enhanced amino acid, lipid, and carbohydrate metabolism compared with the 4-month-old group. Amino acid metabolism is closely related to muscle protein synthesis and growth [34]. In the CAZy analysis, the microbiota of the 8-month-old group exhibited reduced activity in carbohydrate-active enzyme pathways, such as those involving α-amylase, β-galactosidase, and β-glucosidase. These signaling pathways may play key regulatory roles in skeletal muscle development. Siddharth et al. demonstrated that gut microbiota could influence muscle phenotypes and proposed a regulatory mechanism involving the gut–muscle axis [35]. Furthermore, related studies have hypothesized a synchronized gut–brain–muscle axis mediated by microbiota, where rumen fermentation produces large amounts of VFAs and other neurotransmitter precursors. These compounds stimulate the pancreatic nervous center to increase insulin secretion, thereby activating *SREBP1* gene expression and ultimately regulating meat quality [33]. Additionally, SCFAs can directly regulate muscle metabolism and function in mammals through the gut–muscle axis [36]. In this study, significant age-related differences were observed in the ruminal concentrations of propionate, butyrate, and valerate. Butyrate can regulate muscle development by promoting mitochondrial biogenesis and altering gut microbial composition [37,38], while valerate may facilitate the growth of fast-twitch glycolytic muscle fibers by activating the Akt/mTOR pathway [39]. In conclusion, age-induced alterations in the composition, structure, and function of the rumen microbiota in Duolang sheep—particularly in functions such as amino acid metabolism, energy metabolism, and lipid metabolism—lead to variations in VFAs production, which may serve as a potential factor regulating skeletal muscle development.

Metabolomics provides a powerful approach to elucidate the correlations between meat quality phenotypes and muscle metabolites. Furthermore, muscle metabolites can directly influence phenotypic traits such as meat quality and flavor [40]. In this study, metabolomic analysis of the *longissimus thoracis* muscle in Duolang sheep across different ages revealed significant differences in metabolites associated with amino acid metabolism, nucleotide metabolism, and lipid metabolism. The key metabolites involved in amino acid metabolism included betaine, creatine, and glutathione. Betaine serves as a methyl donor, and studies have shown that rumen-protected betaine supplementation in ruminants resulted in a slower post-mortem pH decline and reduced drip loss [41], which aligns with the findings of this study. This may be attributed to slower lactate accumulation after slaughter, which reduces the rate of protein denaturation and thereby decreases moisture loss [42]. Creatine plays a critical role as an energy reservoir in animals, particularly in supporting energy metabolism during skeletal muscle contraction. Research indicates that creatine activates the AMPK signaling pathway, enhancing ATP storage in muscle, reducing glycolytic enzyme activity, slowing post-mortem muscle degradation, and decreasing lactate accumulation [43], which is consistent with our findings. Furthermore, creatine promotes myocyte proliferation by activating the mTOR/p70S6K pathway [44]. In the present study, significant differences in creatine and betaine levels were detected in the 4-month-old group, possibly reflecting rapid skeletal muscle cell proliferation and overall development at this stage. In terms of nucleotide metabolism, significant differences were observed in inosinic acid and L-arginine levels in the 6-month-old group. Inosinic acid plays a key role in imparting umami flavor to meat [45], and previous studies have reported a positive correlation between inosinic acid and sweetness in lamb [46]. Similarly, L-arginine contributes sweet notes and enhances flavor complexity and umami [47]. These findings suggest that the umami and overall flavor quality of 6-month-old Duolang lamb may be superior to that of both 4- and 8-month-old animals. However, several studies have reported that inosinic acid content increased with age, leading to improved meat quality [48], which contrasts with the present results. This discrepancy may be due to species or breed differences, and we hypothesized that 6 months may represent the optimal stage for umami and flavor compound development in Duolang lamb.

The gastrointestinal microbiota and VFAs are closely associated with meat quality and metabolic profiles [36]. In this study, the relative abundances of the class Negativicutes, order Selenomonadales, and family Selenomonadaceae were significantly positively correlated with the b*_45min_ value (yellowness) and the relative content of L-proline in meat. Since L-proline contributes bitterness to meat flavor [49], it is inferred that these microbial taxa may play key roles in regulating meat color and modulating amino acid metabolism. Inosine, a degradation product of inosinic acid, is known to contribute to umami taste and influence meat aroma and flavor formation [50]. In terms of nucleotide metabolism related to flavor, the current study revealed that a negative correlation exists between inosine and the abundance of the genus *Sodaliphilus*, as well as with the ruminal concentrations of propionate, butyrate, and valerate. Conversely, inosinic acid showed significant positive correlations with the relative abundance of the genus *Sodaliphilus* and ruminal butyrate content. These findings indicate that there is a correlation between the aforementioned microorganisms, VFAs, and the flavor development of lamb.

Studies in humans have demonstrated that obesity and skeletal muscle development are associated with the gut microbiota [51], and extensive research has also been conducted on the relationship between the intestinal microbiota and fat deposition in monogastric animals [52]. The rumen is a digestive organ unique to ruminants, harboring a vast community of microorganisms. VFAs, the primary products of microbial fermentation of fibrous materials, serve as the main energy source for the host. Moreover, acetate, propionate, and butyrate are involved in triglyceride synthesis. Additionally, studies have shown that VFAs can influence intramuscular fat deposition and muscle fiber type transformation—thereby affecting meat quality—through G protein-coupled receptors [53], the AMPK signaling pathway [54], the PPAR signaling pathway [55], and the mTOR signaling pathway [56]. In the present study, the relative abundance of rumen microorganisms was significantly correlated with VFA levels, and VFAs were significantly correlated with muscle metabolites such as amino acids and nucleotides. Therefore, it can be inferred that, with increasing age, changes in the composition and function of the rumen microbiota lead to corresponding variations in VFA concentrations, which in turn contribute to differences in muscle metabolite profiles. The specific underlying mechanisms require further investigation using techniques such as transcriptomics.

**Study Limitations:** Although this study comprehensively employed rumen metagenomics and muscle metabolomics to investigate the mechanisms underlying meat quality changes in Duolang sheep at different ages, several limitations must be acknowledged. First, the experimental design included only three age points (4, 6, and 8 months), omitting earlier (e.g., weaning) and later (e.g., finishing phase, adulthood) physiological stages. Consequently, the complete developmental trajectory of meat quality remains unclear. Furthermore, the sample size was modest, comprising 24 lambs in total (*n* = 8 per group). This limited sample size may restrict statistical power and the ability to detect low-abundance metabolites or microbial taxa in high-dimensional multi-omics analyses. Second, while this study identified associations among microbial taxa, metabolites, and meat quality traits through correlation analysis, these correlations do not imply causation. Validation of causal relationships requires further investigation through in vitro culture, gnotobiotic animal models, or controlled intervention studies. Additionally, untargeted metabolomics was primarily utilized for metabolite screening and relative quantification, leaving key differential metabolites (e.g., betaine, inosine monophosphate, L-arginine) without absolute quantitative validation. Future research should incorporate targeted metabolomics to achieve precise quantification of these compounds. Third, regarding generalizability, the study focused exclusively on Duolang sheep, a specific local breed, which necessitates caution when extrapolating findings to other sheep breeds or ruminant species (e.g., goats and cattle). Furthermore, the inclusion of only male lambs precludes the evaluation of sex effects on age-related changes in meat quality. The study was conducted at a single farm under uniform feeding and management conditions, which may not account for variability introduced by different environments, diets, or husbandry practices. Lastly, while associations between rumen microbiota, muscle metabolites, and *longissimus thoracis* quality were observed, the specific molecular pathways through which microbes regulate metabolite production and how these metabolites influence muscle tissue properties remain unclear. It is also uncertain whether similar patterns exist in other muscles (e.g., biceps femoris, psoas major), warranting further investigation. In summary, this study provides valuable insights into the microbial and metabolic mechanisms underlying age-related variations in meat quality. However, future studies incorporating longitudinal designs, integrative multi-omics analyses, functional validation experiments, and larger sample sizes are necessary to corroborate and expand upon these findings.

## 5. Conclusions

In summary, this study demonstrates that the meat quality of the *longissimus thoracis* muscle changes with advancing age, accompanied by shifts in rumen microbial diversity and composition, as well as a trend toward enhanced microbial functional potential. Furthermore, significant correlations were identified among the rumen microbiota, fermentation products, and *longissimus thoracis* muscle metabolites, providing novel evidence to elucidate the molecular mechanisms underlying age-related changes in meat quality and to identify potential biomarkers associated with meat quality traits in this breed. This study suggests that 6 months of age represents the optimal slaughter age for Duolang sheep and that ruminal VFA profiles may serve as a potential biomarker for breeding and quality grading, providing a theoretical basis for regulating meat quality in Duolang sheep.

## Figures and Tables

**Table 1:** Composition and nutrient levels of basic diet (dry matter basis, %).

Dietary Composition	Content	Nutrient Levels ^2^	Content
Corn stalk	45.00	Crude protein	9.88
Alfalfa hay	5.00	Ash	7.91
Corn	30.65	Neutral detergent fiber	41.78
Cottonseed meal	9.25	Acid detergent fiber	22.15
Soybean meal	3.25	Calcium	1.11
Wheat bran	2.60	Phosphorus	0.59
Limestone	0.80	Digestible energy (MJ/kg)	12.69
NaCl	0.45		
CaHPO_4_	0.90		
Premix ^1^	2.00		
Total	100.00		

^1^ The premix provides the following per kg of diet: VA 150,000 IU, VD 20,000 IU, VE 1250 IU, Cu 2500 mg, Mn 1500 mg, Zn 2000 mg, Co 150 mg. ^2^ Except for metabolizable energy, other nutrient levels are measured values.

**Table 2:** Meat quality of *longissimus thoracis* muscle in Duolang sheep at different ages.

Items	Months of Age			SEM	*p*-Value		
	4	6	8		Total	Liner	Quadratic
pH_45min_	6.47	6.80	6.90	0.09	0.12	0.05	0.52
pH_24h_	5.88	6.00	5.95	0.06	0.71	0.63	0.51
L*_45min_	39.08 ^a^	38.99 ^a^	34.80 ^b^	0.76	0.03	0.02	0.17
a*_45min_	14.15	14.85	13.69	0.46	0.60	0.69	0.36
b*_45min_	5.30 ^a^	5.32 ^a^	4.01 ^b^	0.23	0.02	0.02	0.14
L*_24h_	43.31	44.98	41.06	1.08	0.35	0.41	0.24
a*_24h_	15.03	14.46	15.08	0.44	0.83	0.97	0.55
b*_24h_	8.82	9.58	8.95	0.33	0.64	0.88	0.35
Shear force, kgf	8.19	11.92	10.16	0.91	0.26	0.38	0.16
Drip loss rate, %	18.96	19.42	21.54	1.41	0.75	0.48	0.79

^a,b^ Within a row, different letters mean they differed significantly (*p* < 0.05). L*, lightness; a*, redness; b*, yellowness; SEM, standard error of the means.

**Table 3:** The VFAs in the rumen contents of Duolang sheep at different ages (mmol/L).

VFAs	Months of Age			SEM	*p*-Value		
	4	6	8		Total	Liner	Quadratic
Acetate, mmol/L	67.31	71.24	74.46	2.12	0.40	0.18	0.94
Propionate, mmol/L	14.10 ^b^	16.99 ^a^	16.44 ^ab^	0.53	0.06	0.06	0.11
Butyrate, mmol/L	9.47 ^b^	10.89 ^ab^	11.54 ^a^	0.38	0.07	0.03	0.61
Isobutyrate, mmol/L	0.71	0.85	0.79	0.03	0.18	0.28	0.13
Valerate, mmol/L	0.58 ^c^	0.81 ^a^	0.73 ^b^	0.03	0.01	0.05	0.02
Isovalerate, mmol/L	1.22	1.38	1.26	0.05	0.45	0.72	0.23
Total VFAs, mmol/L	93.39	102.17	105.23	2.72	0.19	0.09	0.62

^a,b,c^ Within a row, different letters mean they differed significantly (*p* < 0.05). VFAs, volatile fatty acids; SEM, standard error of the means.

**Table 4:** Alpha diversity indices of rumen microbiota in Duolang sheep at different ages.

Alpha Diversity Index	Months of Age			SEM	*p*-Value		
	4	6	8		Total	Liner	Quadratic
ACE	861.92 ^ab^	941.89 ^a^	829.77 ^b^	19.81	0.05	0.47	0.02
Chao1	878.09 ^ab^	960.59 ^a^	839.77 ^b^	20.44	0.04	0.40	0.02
Shannon	4.22	4.38	4.33	0.04	0.31	0.30	0.26
Simpson	0.86	0.87	0.87	0.01	0.67	0.53	0.53

^a,b^ Within a row, different letters mean they differed significantly (*p* < 0.05); ACE, abundance-based coverage estimator; SEM, standard error of the means.

## Data Availability

The original contributions presented in this study are included in the article. Further inquiries can be directed to the corresponding author.
